# Intracellular osteopontin stabilizes TRAF3 to positively regulate innate antiviral response

**DOI:** 10.1038/srep23771

**Published:** 2016-03-30

**Authors:** Kai Zhao, Meng Zhang, Lei Zhang, Peng Wang, Guanhua Song, Bingyu Liu, Haifeng Wu, Zhinan Yin, Chengjiang Gao

**Affiliations:** 1 Department of Immunology & Key Laboratory of Infection and Immunity of Shandong Province, Shandong University the School of Medicine, Jinan, Shandong 250012, China; 2 The Key Laboratory of Cardiovascular Remodeling and Function Research, Chinese Ministry of Education and Chinese Ministry of Health, Qilu Hospital, Shandong University, Jinan, Shandong, 250012, China; 3 Biomedical Translational Research Institute, Jinan University, Guangzhou, Guangdong, China

## Abstract

Osteopontin (OPN) is a multifunctional protein involved in both innate immunity and adaptive immunity. However, the function of OPN, especially the intracellular form OPN (iOPN) on innate antiviral immune response remains elusive. Here, we demonstrated that iOPN is an essential positive regulator to protect the host from virus infection. OPN deficiency or knockdown significantly attenuated virus-induced IRF3 activation, IFN-β production and antiviral response. Consistently, OPN-deficient mice were more susceptible to VSV infection than WT mice. Mechanistically, iOPN was found to interact with tumor necrosis factor receptor (TNFR)-associated factor 3 (TRAF3) and inhibit Triad3A-mediated K48-linked polyubiquitination and degradation of TRAF3 through the C-terminal fragment of iOPN. Therefore, our findings delineated a new function for iOPN to act as a positive regulator in innate antiviral immunity through stabilization of TRAF3.

The innate immunity is the first line of defense against invading pathogens, which functions to respond to infection directly and relays signals for the activation of the adaptive immunity^1^. During viral infection, multiple signaling pathways in the innate immune system are triggered to promote the production of cytokines to suppress viral replication^2^. Central to the host antiviral response is the production of type I interferons (IFNs), which include IFN-α and IFN-β. Several classes of germline-encoded pattern-recognition receptors (PRRs) have been linked to the production of type I interferons during viral infection. These PRRs include Toll-like receptors (TLRs), retinoic acid–inducible gene I (RIG-I) like receptor (RLRs) and intracellular DNA sensors^3^,^4^,^5^. For example, TLR3 recognizes viral double-stranded RNA in endosomes and triggers a signaling pathway mediated by Toll/IL-1R (TIR) domain-containing adaptor that induces IFN-β (TRIF)^6^. TLR4 also uses TRIF as adaptor to induce IFN-β production^7^. RIG-I is an important cytoplasmic PRR for the detection of positive- and negative-stranded RNA viruses, including Sendai virus (SeV), vesicular stomatitis virus (VSV), hepatitis C virus (HCV), and influenza A virus (IAV)^8^,^9^. The recognition of viral RNA by RIG-I leads to the RIG-I conformation change and the recruitment of the downstream mitochondrial antiviral signaling protein (MAVS) (also called IPS-1, Cardif or VISA) through the CARDs^10^,^11^,^12^,^13^. After recruitment of TRIF and MAVS, TLR3/4 and RIG-I activate convergent pathways composed of tumor necrosis factor receptor (TNFR)-associated factor 3 (TRAF3)^14^, TANK-binding kinase 1 (TBK1)/Iκ-B kinase ε (IKK-ε) and IFN regulatory factor 3 (IRF3), leading to the production of IFN-β^15^. Intracellular DNA from invading pathogens could also induce IFN-β production through a very similar pathway composed of cyclic-GMP-AMP (cGAMP) synthase (cGAS), stimulator of interferon genes protein (STING), TBK1/IKK-ε and IRF3^16^.

TRAF3 is a member of the cytoplasmic signaling protein family called tumor necrosis factor receptor (TNFR)-associated factors (TRAFs), which are composed of 7 members and used by a large and diverse group of receptors including TLR, TNFR and RLR. TRAF3 was first identified to directly associate with CD40 and inhibit CD40-mediated NF-κB activation in B cells^17^,^18^. Subsequent studies demonstrated that TRAF3 is crucial for TLR3/4-induced type I IFN production by macrophages and DCs^19^,^20^. Later on, it was shown that TRAF3 was also involved in the regulation of RLR-induced IFN production^14^,^21^. Activation of TLR3/4 and RLR signaling results in K63-linked polyubiquitylation of TRAF3, which leads to the recruitment and activation of TBK1/IKKɛ and phsophorylation of IRF3. Except for K63-linked polyubiquitination, TRAF3 also undergoes K48-linked ubiquitination by the ubiquitin ligase Triad3A during virus infection, which results in proteasomal degradation of TRAF3 and termination of the type I IFN response^22^. DUBA (deubiquitynating enzyme A) was also found to terminate TLR-induced type I IFN production through cleavage of K63-ubiquitin chains from TRAF3^23^. But, the detailed regulatory mechanism for TRAF3 polyubiquitination is not clear.

Osteopontin (OPN), encoded by gene *Spp1*, is a secreted glycoprotein which regulates diverse biological processes including differentiation, adhesion, bone remodeling, malignancy and immune response. Recently, it was demonstrated that there are two forms of OPN: secreted OPN (sOPN) and intracellular OPN (iOPN), which are translated from different start codon in the single OPN mRNA^24^. Compared to sOPN, iOPN lost the N-terminal signal sequence, which targets OPN to the secretory system. Therefore, iOPN is mainly localized in the cell. However, the function of iOPN in innate antiviral immune response is not known.

In this study, we provided evidence to demonstrate that iOPN is an essential positive regulator in the innate antiviral immunity. OPN expression is enhanced by VSV and SeV infection. Knockdown of OPN significantly attenuated virus-induced IFN-β production and enhanced VSV replication, while overexpression of iOPN showed an opposite effect. OPN-deficient mice showed less IFN-β production and increased VSV replication, which further confirmed the positive role of OPN in antiviral response. iOPN was found to interact with and inhibit K48-linked polyubiquitination and degradation of TRAF3. Therefore, our study identified iOPN as a positive regulator in innate antiviral signaling through stabilization of TRAF3.

## Results

### OPN expression is induced upon virus infection

**Figure 1:**
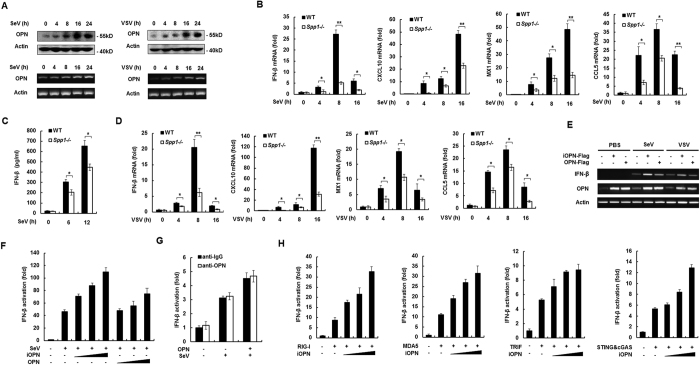
OPN positively regulates IFN-β production. (**A**) Western blot and RT-PCR analysis of OPN expression in mouse peritoneal macrophages infected with SeV or VSV for indicated times. (**B**) Quantitative RT-PCR analysis of IFN-β, CXCL10, Mx1 and CCL5 mRNA in peritoneal macrophages from WT and OPN-deficient (*Spp1*^−/−^) mice infected with SeV for indicated time periods or left uninfected. (**C**) ELISA analysis of IFN-β secretion in peritoneal macrophages from WT and OPN-deficient (*Spp1*^−/−^) mice infected with SeV for indicated time periods. (**D**) Quantitative RT-PCR analysis of IFN-β, CXCL10, MX1 and CCL5 mRNA in peritoneal macrophages from WT and OPN-deficient (*Spp1*^−/−^) mice infected with VSV for indicated time periods or left uninfected. (**E**) RT-PCR analysis of IFN-β mRNA expression in HEK293 cells transfected with full length OPN and iOPN expression plasmids for 24 h, then infected with SeV, VSV for 8 h or left uninfected. (**F**) Luciferase activity in HEK293 cells transiently transfected with IFN-β promoter reporter together with increasing amount of iOPN and full length OPN expression plasmids for 24 h, and then infected with SeV for 8 h. (**G**) Luciferase activity in HEK293 cells transfected with IFN-β promoter reporter and full length OPN expression plasmid or control vector for 24 h, then the cells were pretreated with OPN specific antibody or control IgG for 4 h, and infected with SeV for 16 h. (**H**) Luciferase activity in HEK293 cells transfected with expression plasmids for IFN-β promoter reporter, RIG-I, MDA5, TRIF or STING+cGAS together with increasing amount of iOPN plasmid. The data are representative of three biological replicates (mean ± S.D. in **B–D** and **F–H**). **P < 0.01; *P < 0.05.

To investigate whether OPN is involved in antiviral response, OPN expression was measured in murine peritoneal macrophages infected with Sendai virus (SeV) and vesicular stomatitis virus (VSV), which have been shown to trigger the RIG-I signaling. The expression of OPN protein and mRNA was increased after infection with SeV (Fig. 1A). Similarly, infection of VSV also increased OPN protein and mRNA expression in peritoneal macrophages (Fig. 1A). ELISA analysis showed that sOPN was also increased upon virus infection (Fig. S1A). Similar to SeV infection, activation of the intracellular DNA receptor signaling by ISD (interferon-stimulating DNA) and cGAMP (cyclic GMP-AMP) induced OPN expression (Fig. S1B). Consistently, infection with a DNA virus Herpes simplex virus-1 (HSV-1) also increased OPN protein expression (Fig. S1C) All together, these data demonstrated that OPN expression is induced by virus infection in murine peritoneal macrophages.

### OPN positively regulates IFN-β production

To investigate the function of OPN in innate antiviral immune response, peritoneal macrophages were prepared from WT and OPN-deficient (*Spp1*^−/−^) mice and infected with SeV for various times. Then, the expression of IFN-β was measured. SeV infection induced the expression of IFN-β mRNA in WT macrophages. However, SeV-induced IFN-β mRNA expression was greatly decreased in OPN-deficient macrophages compared to that in WT macrophages (Fig. 1B). Consistently, OPN-deficient macrophages secreted less IFN-β protein than WT macrophages after SeV infection (Fig. 1C). The expression of CXCL10, Mx1 and CCL5, which are downstream genes of IFN-β signaling pathway, also decreased in SeV-infected OPN-deficient macrophages (Fig. 1B). Similar to SeV infection, VSV infection-induced expression of IFN-β, CXCL10, Mx1 and CCL5 also greatly decreased in OPN-deficient macrophages (Fig. 1D). LPS and poly(I:C) (polyinosinic: polycytidylic acid) stimulation, which activate TLR4 and TLR3 signaling respectively, induced less IFN-β production in OPN-deficient macrophages compared to that in WT macrophages (Fig. S2A). ISD transfection, which was shown to activate intracellular DNA receptors signaling, also led to a decreased IFN-β production in OPN-deficient macrophages compared to that in WT macrophages (Fig. S2B).

We further investigated the function of OPN on IFN-β expression using overexpression experiments. Transfection of iOPN and full length OPN expression plasmids into HEK293 cells increased SeV- and VSV-induced IFN-β expression (Fig. 1E). Similar to IFN-β mRNA expression, transfection of iOPN and full length OPN expression plasmids also increased SeV-induced IFN-β promoter activation in a dose-dependent manner (Fig. 1F). Further, we found that addition of OPN antibody into the culture medium could not inhibit full length OPN transfection-mediated IFN-β activation induced by SeV infection (Fig. 1G). Overexpression of iOPN also increased RIG-I-, melanoma differentiation-associated gene 5 (MDA5)-, TRIF- and cGAS+STING-induced IFN-β promoter activation in a dose-dependent manner (Fig. 1H). In all circumstances, intracellular OPN seemed more potent to induce IFN-β expression than the full length OPN. Taken together, these data indicated that OPN, especially iOPN, positively regulates IFN-β production downstream of various innate immune signaling pathways including TLR3/4, RLRs and intracellular DNA receptor signaling.

### OPN potentiates antiviral response

**Figure 2:**
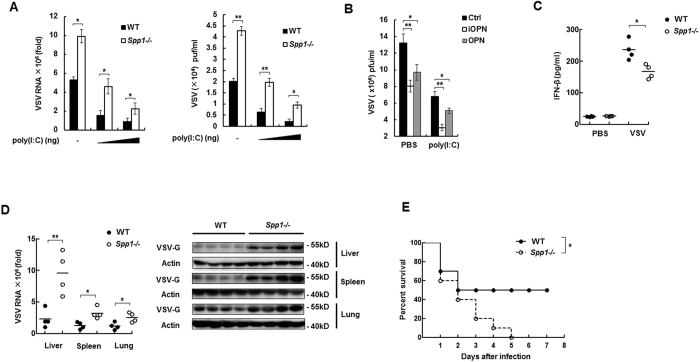
OPN potentiates antiviral response. (**A**) Mouse peritoneal macrophages from WT and *Spp1*^−/−^ mice were transfected with different amount of poly(I:C) (50 ~ 100 ng) for 18 h, followed by infection with VSV (MOI, 0.1) for 12 h. Intracellular VSV RNA replicates (left) and VSV titers (right) were measured by quantitative RT-PCR and standard plaque assay, respectively. (**B**) HEK293 cells were first transfected with iOPN or full length OPN expression plasmid for 24 h, then the cells were transfected with 100ng poly(I:C), followed with VSV infection. VSV titers were measured as in (**A**). (**C**) ELISA analysis of IFN-β production in sera from WT and *Spp1*^−/−^ mice (n = 4 per group) intraperitoneally infected with VSV for 24 h (1 × 10^8 ^pfu per mouse). (**D**) QRT-PCR analysis of VSV RNA replication (left) and western blot of VSV-G protein (right) in liver, spleen and lung from WT and *Spp1*^−/−^ mice which were intraperitoneally infected with VSV for 72 h (1 × 10^8 ^pfu per mouse). (**E**) Survival of WT and *Spp1*^−/−^ mice intraperitoneally infected with VSV (5 × 10^8 ^pfu per mouse) (n = 10 per group). Data are representative of four (**A,B**) or three (**C–E**) biological replicates (mean ± S.D. in **A–E**). **P < 0.01; *P < 0.05.

IFN-β plays an essential role in antiviral immune response^25^. To investigate the role of OPN in antiviral response, VSV was used to infect cells. Plaque assays showed that VSV replication greatly increased in peritoneal macrophages prepared from OPN-deficient mice compared to that from WT mice in the presence or absence of poly(I:C) (Fig. 2A). Consistently, VSV RNA was also increased in OPN-deficient macrophages compared to that in WT macrophages (Fig. 2A). In contrast, overexpression of iOPN and the full length OPN in HEK293 cells greatly attenuated VSV replication in the presence or absence of poly(I:C) (Fig. 2B). Taken together, these data indicated that OPN positively regulates antiviral immune response.

To investigate the physiological role of OPN in antiviral response *in vivo*, *Spp1*^−/−^ mice and WT mice were infected with VSV, and the antiviral immune responses were examined. The amount of IFN-β protein induced by VSV infection was much less in sera of VSV-infected *Spp1*^−/−^ mice than that of WT mice (Fig. 2C). In accordance with reduced IFN-β production, VSV replication in the livers, spleens, and lungs was much higher in OPN-deficient (*Spp1*^−/−^) mice than in WT controls (Fig. 2D). Importantly, *Spp1*^−/−^ mice were more susceptible to VSV infection than WT mice (Fig. 2E). *Spp1*^−/−^ mice all died, while 50% of WT mice were alive 5 days after infection. These data suggested that OPN is an important positive regulator of IFN-β production and antiviral immune responses.

### iOPN positively regulates IRF3 activation

**Figure 3:**
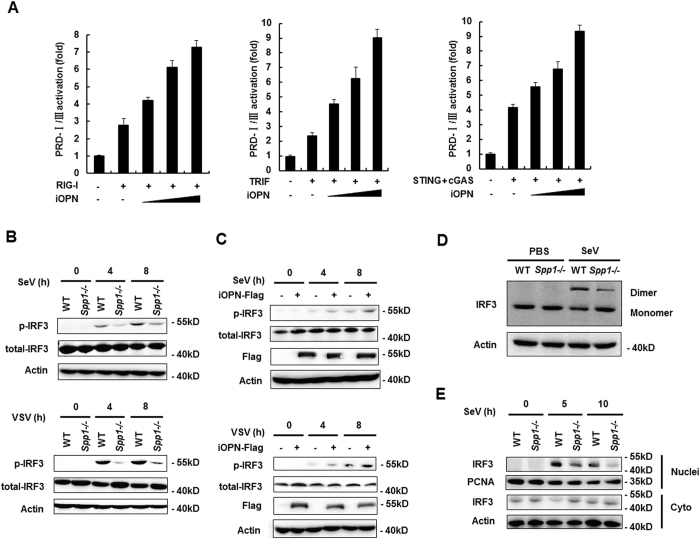
iOPN positively regulates IRF3 activation. (**A**) Luciferase activity in HEK293 cells transiently transfected with IFN-β PRDI/III reporter plasmid and expression plasmids for RIG-I, TRIF and cGAS+STING together with increasing amount of iOPN expression plasmid for 24 h. (**B**) Western blot of phosphorylated IRF3 and total IRF3 in WT and OPN-deficient peritoneal macrophages infected with SeV or VSV for indicated times. (**C**) HEK293 cells were transfected with iOPN expression plasmid or control vector for 24 h, then infected with SeV or VSV for indicated times, phosphorylated IRF3 and total IRF3 were analyzed by western blot. (**D**) WT and OPN-deficient (*Spp1*^−/−^) peritoneal macrophages were infected with SeV for 8 h or left uninfected. Cell lysate were separated by native PAGE and analyzed by western blot with the indicated antibodies. (**E**) The nuclear and cytoplasmic fractions were prepared from WT and OPN-deficient (*Spp1*^−/−^) peritoneal macrophages infected with SeV for indicated times, then western blot were performed with the indicated antibodies. The data are representative of three biological replicates (mean ± S.D. in **A**).

IRF3 is the main transcription factor responsible for IFN-β transcription during the early phase of viral infection^26^. To investigate the function of OPN on IRF3 activation, series of experiments were performed. First, IFN-β PRD I/III reporter, which harbors only IRF3 binding site in IFN-β promoter, was used^27^. RIG-I, TRIF- and cGAS+STING-induced-IFN-β PRD I/III activation was increased by iOPN overexpression in a dose-dependent manner (Fig. 3A). IRF3 activation requires the phosphorylation of conserved serine and theronine residues at the c-terminal region^28^. SeV infection induced IRF3 phosphorylation in macrophages from WT mice (Fig. 3B). While, SeV-induced IRF3 phophorylation was greatly decreased in macrophages from OPN-deficient (*Spp1*^−/−^) mice (Fig. 3B). Similarly, VSV infection-induced IRF3 phosphorylation was also greatly decreased in macrophages from *Spp1*^−/−^ mice compared to that from WT mice (Fig. 3B). LPS- and poly(I:C)-induced IRF3 phosphorylation was similarly decreased in macrophages from *Spp1*^−/−^ mice compared to that from WT mice (Fig. S3A and B). In contrast, overexpression of iOPN in HEK293 cells substantially increased SeV- and VSV-induced IRF3 phosphorylation (Fig. 3C). After phosphorylation, IRF3 dimerizes and translocates into nuleus to initiate IFN-β transcription^28^. IRF3 dimerization was greatly decreased in OPN-deficient (*Spp1*^−/−^) macrophages compared to that in WT macrophages after SeV infection (Fig. 3D). Western blot analysis of cytoplasmic fraction and nuclear fraction showed that more IRF3 was translocated into nucleus in macrophages from WT mice compared to that from OPN-deficient (*Spp1*^−/−^) mice after virus infection (Fig. 3E). All together, these data demonstrated that OPN positively regulates IRF3 activation to regulate IFN-β production and antiviral response.

### iOPN interacts with TRAF3

**Figure 4:**
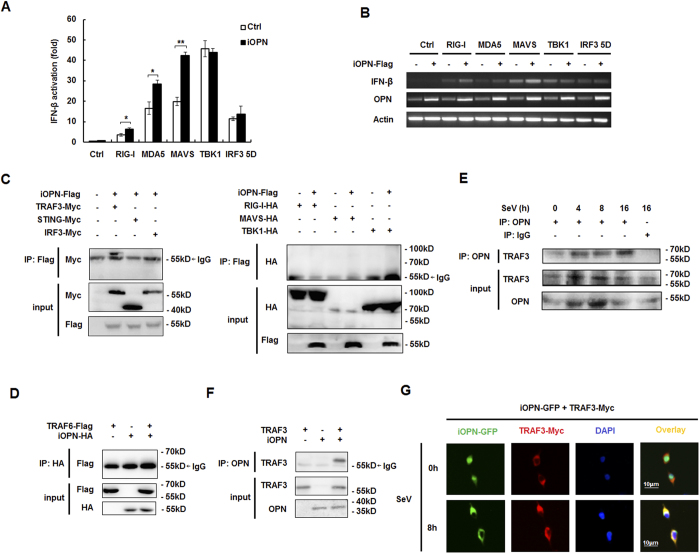
iOPN interacts with TRAF3. (**A**) Luciferase activity in HEK293 cells transfected with expression plasmids for RIG-I, MDA5, MAVS, TBK1 or IRF3 5D and IFN-β promoter reporter along with iOPN expression plasmid. (**B**) RT-PCR analysis of IFN-β expression in HEK293 cells transfected with expression plasmids for RIG-I, MDA5, MAVS, TBK1 or IRF3 5D and iOPN expression plasmid. (**C**) HEK293 cells were transfected with Flag-iOPN together with expression plasmids for Myc-TRAF3, Myc-STING, Myc-IRF3, HA-RIG-I, HA-MAVS or HA-TBK1, followed by immunoprecipitation (IP) with anti-Flag antibody and western blot with anti-Myc antibody or anti-HA antibody. Input, western blot of whole-cell lysate with indicating antibodies. (**D**) HEK293 cells were transfected with HA-iOPN expression plasmid together with expression plasmid for Flag-TRAF6, followed by IP with anti-HA antibody and western blot with anti-Flag antibody. Input, western blot of whole-cell lysate with indicating antibodies. (**E**) Mouse peritoneal macrophages were infected with SeV for various times, followed by immunoprecipitation (IP) with OPN antibody or control IgG and western blot with anti-TRAF3 antibody. Input, western blot of whole-cell lysate with indicating antibodies. (**F**) TRAF3 and iOPN were obtained by *in vitro* transcription and translation. Interaction between TRAF3 and iOPN was assayed by mixing TRAF3 and iOPN together followed by IP with OPN antibody and western blot with anti-TRAF3 antibody. (**G**) Fluorescent images of HEK293 cells transfected with GFP-iOPN together with Myc-TRAF3. Nuclei and Myc-TRAF3 were labeled with DAPI (blue) and antibody to Myc tag (red), respectively. The data are representative of three biological replicates (mean ± S.D. in **A**). **P < 0.01; *P < 0.05.

OPN positively regulates IFN-β production downstream of TLR3/4, RLR and intracellular DNA receptor signaling pathways, indicating OPN targets common molecules in these signaling pathways. To identify the molecules, IFN-β promoter activation induced by various molecules in RLRs signaling pathway was investigated. We found iOPN increased RIG-I-, MDA5-, MAVS-induced IFN-β activation, but not TBK1- and IRF3 5D-induced IFN-β activation (Fig. 4A). RT-PCR analysis of IFN-β mRNA also confirmed iOPN increased RIG-I-, MDA5-, MAVS-induced IFN-β expression, but not TBK1- and IRF3-5D-induced IFN-β expression (Fig. 4B). These data indicated that iOPN targets molecules upstream of TBK1 to positively regulate innate signaling.

To directly identify iOPN targets, immunoprecipitation (IP) and western blotting (WB) were performed in HEK293 cells transfected with expression plasmids for RIG-I, MAVS, TRAF3, TNF receptor-associated factor 6 (TRAF6), STING, TBK1 and IRF3 together with iOPN. As shown in Fig. 4C, iOPN was shown to interact with TRAF3, but not with RIG-I, MAVS, STING, TBK1 and IRF3. TRAF3 has been shown to be required for the IFN-β expression downstream of TLR3/4 and RLR signaling^19^,^20^. Interestingly, iOPN could not interact with TRAF3 homologue TRAF6, which has been shown to activate NF-κB, leading to the production of proinflammatory cytokines (Fig. 4D). Interaction between endogenous TRAF3 and OPN also detected in macrophages after SeV infection (Fig. 4E).

To confirm iOPN interacts with TRAF3 directly, iOPN and TRAF3 were expressed in an *in vitro* protein expression system, then mixed together and followed by pull-down assays with anti-OPN antibody. As shown in Fig. 4F, TRAF3 could coimunoprecipitate with OPN, indicating a direct interaction between iOPN and TRAF3.

The interactions were further supported by the colocalization studies. iOPN-GFP was found to diffuse in the cytoplasm and nucleus without SeV infection (Fig. 4G). TRAF3 was present in the cytoplasm exclusively (Fig. 4G). iOPN-GFP and TRAF3 showed less or no colocalization without SeV infection (Fig. 4G). SeV infection induced translocation of large amount of iOPN from nucleus into cytoplasm, where colocalization between iOPN and TRAF3 was greatly increased (Fig. 4G). Taken together, these data suggested that iOPN interacts with TRAF3 to positively regulate IFN-β production and antiviral response.

### iOPN inhibits K48-linked polyubiquitination and degradation of TRAF3

**Figure 5:**
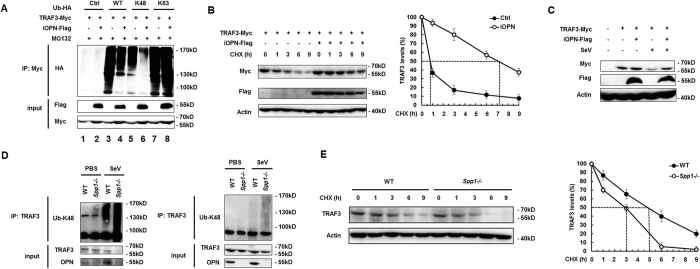
iOPN inhibits K48-linked polyubiquitination and degradation of TRAF3. (**A**) Lysate from HEK293 cells transiently transfected with expression plasmids for Myc-TRAF3, Flag-iOPN and HA-Ub (WT), HA-Ub (K48) or HA-Ub (K63) were subjected to IP with anti-Myc antibody, followed by western blot with anti-HA antibody. Input, western blot of whole-cell lysate with indicating antibodies. (**B**) Western blot of Myc-TRAF3 protein in lysate of HEK293 cells transfected with Myc-TRAF3 and Flag-iOPN expression plasmids for 24 h, then infected with SeV for 2 h followed by treatment with cycloheximide (CHX, 100 μg/ml) for indicated times (left). The relative amount of total TRAF3 was quantified by densitometry and plotted with respect to treatment time. The values were normalized to actin. TRAF3 level at time 0 h was defined as 100%, the dashed line indicates the TRAF3 half-life where 50% of the TRAF3 protein level was reached (right). (**C**) A549 cells transfected with Myc-TRAF3 and Flag-iOPN expression plasmids or control vector were infected with SeV for 8 h, then western blot was performed with indicated antibodies. (**D**) Lysate from WT and OPN-deficient (*Spp1*^−/−^) peritoneal macrophages infected with SeV for 8 h were subjected to immunoprecipitation under stringent condition (right) or normal condition (left) with anti-TRAF3 antibody followed by western blot with anti-Ub K48 antibody. (**E**) Western blot of TRAF3 protein in lysate of WT and OPN-deficient (*Spp1*^−/−^) macrophages infected with SeV for 2 h, then treated with CHX (100 μg/ml) for indicated times (left). The relative amount of total TRAF3 was quantified by densitometry and plotted with respect to treatment time. The values were normalized to actin. TRAF3 level at time 0 h was defined as 100%, the dashed line indicates the TRAF3 half-life where 50% of the TRAF3 protein level was reached (right). The data are representative of three biological replicates.

TRAF3 activation is tightly regulated by protein ubiquitination. K63-linked TRAF3 polyubiquitination is responsible for the activation of downstream signaling^29^. While, K48-linked ubiquitination leads to the degradation of TRAF3 and deactivation of TRAF3-mediated downstream signaling^22^,^29^. To investigate the molecular mechanism of iOPN in the regulation of IFN-β production, TRAF3 polyubiquitination was investigated. TRAF3 was transfected into HEK293 cells together with WT HA-ubiquitin plasmid and iOPN expression plasmid. IP and WB showed that TRAF3 polyubiquitination was greatly inhibited by iOPN expression (Fig. 5A, lane 4 vs. lane 3). To investigate which form of TRAF3 polyubiquitination was affected by iOPN, HA-ubiquitin mutants K48 and K63, which has only one lysine residue at position 48 and 63 respectively, were transfected into HEK293 cells together with iOPN expression plasmid. Overexpression of iOPN decreased TRAF3 polyubiquitination in HA-K48-transfected cells (Fig. 5A, lane 6 vs. lane 5), whereas, TRAF3 polyubiquitination was not affected by iOPN in HA-K63-transfected cells (Fig. 5A, lane 8 vs. lane 7), indicating iOPN mainly prevents TRAF3 from K48-linked polyubiquitination.

K48-linked polyubiquitination leads to protein degradation by 26S proteasome. To investigate the function of iOPN on TRAF3 degradation, Myc-TRAF3 was transfected into HEK293 cells together with iOPN expression plasmid or control plasmid. After SeV infection, the half life of Myc-TRAF3 protein was measured. SeV infection led to the degradation of Myc-TRAF3 in control vector transfected cells with a half life of ~0.8 h (Fig. 5B). However, the degradation of Myc-TRAF3 was greatly attenuated in iOPN expression vector transfected cells (Fig. 5B, 7.2 h vs. 0.8 h). Similarly, SeV infection induced the degradation of Myc-TRAF3 in A549 cells, whereas, overexpression of iOPN reversed Myc-TRAF3 protein degradation after SeV infection (Fig. 5C), indicating inhibition of TRAF3 degradation by iOPN is not cell specific.

To further confirm OPN stabilizes TRAF3 protein through inhibition of K48-linked ubiquitination in physiological conditions, peritoneal macrophages from WT and *Spp1*^−/−^ mice were prepared and infected with SeV. IP and WB showed that K48-linked polyubiquitination of TRAF3 was greatly increased in the macrophages from *Spp1*^−/−^ mice compared to that in macrophages from WT mice after SeV infection (Fig. 5D, left). Increased K48-linked TRAF3 polyubiquitination in OPN-deficient macrophages was further confirmed with proteins immunoprecipitated with anti-TRAF3 under stringent conditions (Fig. 5D, right). Consistent with more TRAF3 ubiquitination, TRAF3 was degraded more rapidly in OPN-deficient macrophages (Fig. 5E, 5 h vs. 3 h). All together, these data indicated that OPN prevents TRAF3 from K48-linked polyubiquitination and degradation.

### iOPN inhibits Trid3A-mediated TRAF3 polyubiquitination

**Figure 6:**
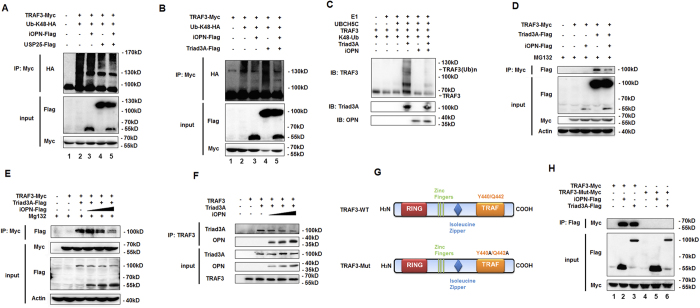
iOPN inhibits Triad3A-mediated TRAF3 polyubiquitination. (**A**) Lysate from HEK293 cells transiently transfected with expression plasmids for Myc-TRAF3, HA-Ub (K48) or Flag-iOPN and Flag-USP25 were subjected to IP with anti-Myc antibody, followed by western blot with anti-HA antibody. Input, western blot of whole-cell lysate with indicating antibodies. (**B**) Lysate from HEK293 cells transiently transfected with expression plasmids for Myc-TRAF3, HA-Ub (K48) or Flag-iOPN and Flag-Triad3A were subjected to IP with anti-Myc antibody, followed by western blot with anti-HA antibody. Input, western blot of whole-cell lysate with indicating antibodies. (**C**) TRAF3, Triad3A, iOPN protein were obtained by *in vitro* transcription and translation. *In vitro* ubiquitination assay was performed in the presence of Ub, E1, UbcH5c, iOPN, Triad3A and TRAF3. The ubiquitination of TRAF3 was examined by WB with TRAF3 antibody. (**D**,**E**) HEK293 cells were transfected with Myc-TRAF3, together with expression plasmids for Flag-Traid3A and Flag-iOPN (**D**) or increasing amount of iOPN expression plasmid (**E**) followed by IP with anti-Myc antibody and western blot with anti-Flag antibody. Input, western blot of whole-cell lysate with indicating antibodies. (**F**) TRAF3, Triad3A, iOPN protein were obtained by *in vitro* transcription and translation. *In vitro* pull-down assays was performed in the presence of TRAF3, Triad3A and increasing amount of iOPN protein followed by IP with anti-TRAF3 antibody and western blot with indicated antibodies. (**G**) Schematic structure of TRAF3-WT and TRAF3-Mut. In TRAF3-Mut, the residues at position 441 and 443 were substituted with alanine. (**H**) Lysate from HEK293 cells transiently transfected with expression plasmids for Myc-TRAF3-WT or Myc-TRAF3-Mut together with Flag-iOPN or Flag-Triad3A plasmid were subjected to IP with anti-Flag antibody, followed by western blot with anti-Myc antibody. Input, western blot of whole-cell lysate with indicating antibodies. The data are representative of three biological replicates.

iOPN alone does not have the ability to modulate protein ubiquitination. There are two possibilities for iOPN to inhibit TRAF3 polyubiquitination. One is that iOPN may recruit deubiquitinating enzymes (DUB) to cleave K48-linked polyubiquitin chains from TRAF3 to stabilize TRAF3. Recently, USP25 has been reported to cleave K48-linked polyubiquitin chains from TRAF3^30^,^31^. To investigate whether iOPN recruit USP25 to stabilize TRAF3, TRAF3 ubiquitination was measured in the presence of USP25 and iOPN expression plasmids. iOPN expression decreased TRAF3 ubiquitination (Fig. 6A, lane 2). USP25 expression indeed decreased TRAF3 polyubiquitination (Fig. 6A, lane 3). While, cotransfection of USP25 and iOPN could not further decrease USP25-mediated deubiquitination from TRAF3 (Fig. 6A, lane 4), suggesting iOPN may not recruit USP25 to cleave K48-linked ubiquitin from TRAF3.

Another possibility is that iOPN prevents an E3 ligase from binding to TRAF3. Triad3A has been reported to be an E3 ligase involved in TRAF3 ubiquitination and degradation after virus infection^22^. To investigate whether iOPN inhibits Triad3A-mediated TRAF3 ubiquitination, TRAF3 was transfected into HEK293 cells together with Triad3A and iOPN. IP and WB showed that Triad3A promoted TRAF3 ubiquitination (Fig. 6B, lane 4 vs. lane 2). Overexpression of iOPN greatly decreased TRAF3 ubiquitination mediated by Triad3A (Fig. 6B, lane 5). *In vitro* ubiquitination assays with *in vitro* expressed proteins also confirmed that Triad3A-induced K48-linked TRAF3 polyubiqutination was greatly attenuated by iOPN (Fig. 6C). Triad3A binding to TRAF3 was also decreased by iOPN in a dose-dependent manner (Fig. 6D,E). *In vitro* pull-down assays confirmed the binding Triad3A to TRAF3 was gradually decreased with the increasing binding of iOPN to TRAF3 (Fig. 6F). Consistent with the inhibition of Triad3A-induced TRAF3 ubiqutination, Triad3A-induced degradation of TRAF3 was reversed by iOPN expression (Fig. 6B, input, lane 5 vs. lane 4). The Y residue and Q residue at position 441 and 443 of TRAF3 have been reported for Triad3A binding^22^. To confirm the importance of these two residues, TRAF3 mutant was constructed by mutating Y441 and Q443 to A (Fig. 6G). Mutation of YQ to AA ablated Triad3A binding to TRAF3 (Fig. 6H, lane 6 vs. lane 3). Notably, iOPN binding to TRAF3 mutant was also ablated, indicating iOPN binding to the same sites in TRAF3 as the Triad3A (Fig. 6H, lane 5 vs. 2). All together, these data demonstrated that iOPN compete with Trida3A for the binding to TRAF3, which prevents TRAF3 from K48-linked polyubiquitination and degradation promoted by Triad3A.

### C-terminal fragment of iOPN binds to TRAF3

**Figure 7:**
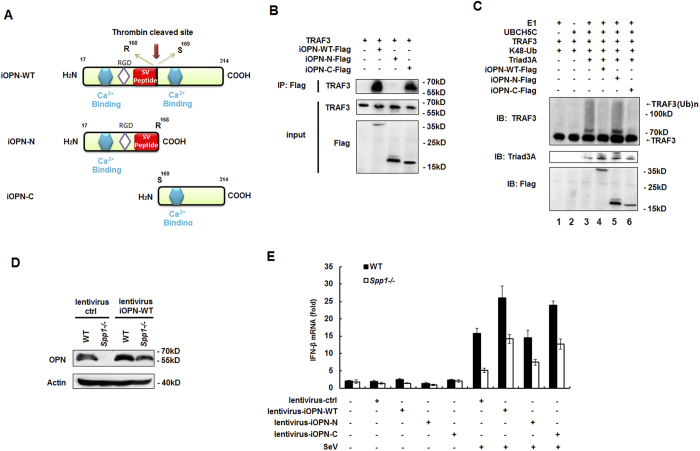
C-terminal fragment of iOPN binds to TRAF3. (**A**) Schematic structure of iOPN-WT and its mutant iOPN-N and iOPN-C containing the N-terminal and C-terminal fragment of iOPN divided in the thrombin cleaved site. (**B**) TRAF3, Flag-iOPN-WT, Flag-iOPN-N, Flag-iOPN-C protein were obtained by *in vitro* transcription and translation. Interaction between TRAF3 and iOPN truncations was assayed by mixing TRAF3 and iOPN truncations together followed by IP with Flag antibody and western blot with TRAF3 antibody. (**C**) TRAF3, Triad3A, Flag-iOPN-WT, Flag-iOPN-N, Flag-iOPN-C protein were obtained by *in vitro* transcription and translation. *In vitro* ubiquitination assays were performed in the presence of Ub, E1, UbcH5c, TRAF3, Triad3A and iOPN-WT, iOPN-N or iOPN-C. The ubiquitination of TRAF3 was examined by western blot with TRAF3 antibody. (**D**) Peritoneal macrophages from WT and OPN-deficient (*Spp1*^−/−^) mice which were infected with lentivirus (MOI, 50) containing iOPN-WT expression plasmid or control plasmid, 4 days later, OPN protein level were assayed by using WB. (**E**) Peritoneal macrophages from WT and OPN-deficient (*Spp1*^−/−^) mice were infected with lentivirus (MOI, 50) containing control vector, iOPN-WT, iOPN-N or iOPN-C for 4 days, then infected with SeV for 8 h or left uninfected, qRT-PCR analysis of IFN-β was performed. The data are representative of three biological replicates (mean ± S.D. in **E**).

Endogenous OPN can be cleaved by thrombin at position 168 into two fragments^32^. In order to investigate the OPN fragment involved in the binding and regulation of TRAF3 ubiquitination, two OPN truncations were constructed and expressed *in vitro* (Fig. 7A). *In vitro* pull-down assays demonstrated that full length and the C-terminal fragment of iOPN, but not the N-terminal fragment, bound to TRAF3 (Fig. 7B). Consistent with the C-terminal fragment binding to TRAF3, Trida3A-induced TRAF3 ubiqutination was inhibited by the C-terminal fragment (lane 6), but not the N-terminal fragment (Fig. 7C, lane 5). These data indicated that the C-terminal fragment of OPN is responsible for the binding and inhibition of ubiquitination of TRAF3.

To investigate the inhibition of TRAF3 ubiquitination by WT and the C-terminal fragment of iOPN has a physiological role on IFN-β production, lentiviral expression plasmids for WT, N-terminal fragment and C-terminal fragment of iOPN were constructed and used to infect WT and OPN-deficient macrophages. Infection of lentivirus containing WT iOPN plasmid increased OPN expression in WT macrophages and restored iOPN expression in OPN-deficient (*Spp1*^−/−^) macrophages (Fig. 7D). Consistent with positive function of iOPN on IFN-β production, lentiviral infection of WT iOPN expression plasmid into WT macrophages further increased SeV-induced expression of IFN-β, CXCL10, Mx1 and CCL5 (Figs 7E and S4). Lentiviral infection of WT iOPN expression plasmid into OPN-deficient macrophages restored SeV-induced expression of IFN-β, CXCL10, Mx1 and CCL5 to the same level as that in WT macrophages (Figs 7E and S4). Consistent with ability to inhibit TRAF3 ubiquitination by the C-terminal fragment, lentiviral infection of the C-terminal fragment of iOPN increased SeV-induced expression of IFN-β, CXCL10, Mx1 and CCL5 in WT macrophages (Figs 7E and S4). SeV-induced expression of IFN-β, CXCL10, Mx1 and CCL5 in OPN-deficient macrophages was also restored upon infection with lentivirus containing the C-terminal fragment of iOPN (Figs 7E and S4). But, infection of lentivirus containing the N-terminal fragment of iOPN could not increase or restore SeV-induced expression of IFN-β, CXCL10, Mx1 and CCL5 in WT and OPN-deficient macrophages, respectively (Figs 7E and S4). Taken together, these data demonstrated that iOPN binds to TRAF3 through the C-terminal fragment, preventing TRAF3 from K48-linked ubiquitination and degradation and leading to increased IFN-β production and innate antiviral response.

## Discussion

Osteopontin (OPN) is a multifunctional protein involved in both innate immunity and adaptive immunity. However, the function of OPN in antiviral immune responses remains controversial. Brown *et al*. showed that knockdown of OPN in primary human macrophages resulted in decrease in HIV-1 replication and ectopic expression of OPN significantly enhanced HIV infectivity and replication, suggesting that OPN facilitates HIV replication^33^. While, Sato *et al*. reported that OPN-deficient mice exhibit higher virus titer and poor survival data after the lethal infection of influenza A virus (IAV) compared to the wild type (WT) mice and OPN transgenic mice, suggesting that OPN plays an important role in host defense against IAV infection^34^. Furthermore, Abel *et al*. demonstrated that OPN is not required for the host against infection of vaccinia virus strain WR (VV-WR) or influenza virus strain PR8 using OPN-deficient mice^35^. In the present study, we provided evidence to demonstrate that OPN plays essential roles in regulating innate antiviral immunity. Knockdown of OPN expression attenuated virus-induced IFN-β production and enhanced VSV replication, while overexpression of OPN increased virus-induced IFN-β production and attenuated VSV replication. Furthermore, OPN-deficient mice showed less IFN-β production, increased VSV replication and more susceptible to VSV infection.

OPN has two isoforms, sOPN and iOPN, which are initiated from one single mRNA but from different start codon^24^. The function of sOPN on the immune regulation has been studied extensively. But, the function of iOPN is still largely unknown. Recently, iOPN has been demonstrated to play essential roles in TLR signaling. For example, TLR9 stimulation promotes association between iOPN and MyD88, leading to IRF7 activation and IFN-α production in pDCs^36^. iOPN is also reported to be involved in TLR2 and dectin-1 pathways^37^. iOPN was identified to work as an adaptor molecule to facilitate formation of a receptor cluster composed of TLR2, dectin-1 and mannose receptor that are involved in anti-fungal responses. At the same time, iOPN associates with signaling molecules IRAK1 and Syk downstream of the TLR2 pathway and dectin-1 pathway, respectively, resulting in the MAPK activation.

In this study, we found transfection of iOPN expression plasmid could induce IFN-β production, IRF3 activation and attenuate VSV replication. At the same time, we found transfection of the full length OPN expression plasmid could also induce IFN-β production, IRF3 activation and attenuate VSV replication. However, full length OPN expression plasmid was found to be less potent to induce IFN-β production compared to iOPN expression plasmid. Furthermore, we found that addition of OPN antibody into the culture medium could not inhibit full length OPN transfection-mediated IFN-β activation induced by SeV infection, indicating that the secrted form OPN is not involved in the positive regulation of IFN-β signaling. Thus, the phenomenon that full length OPN expression plasmid induced IFN-β production may be caused by the translation and production of iOPN from the unknown translation start site in the full length OPN expression plasmid. Alternatively, it may be contributed to the C-terminal fragment of full length OPN cleaved by thrombin because we demonstrated that C-terminal fragment of iOPN could efficiently restored the OPN function in OPN-deficient macrophages. All together, our data suggested that iOPN is an important positive regulator of innate antiviral immunity.

We found that iOPN interacted with TRAF3 after virus infection. OPN is an extremely acidic protein. When it is overexpressed, OPN may bind non-specifically to some, especially basic, intracellular proteins. We found overexpressed iOPN could not interact with the TRAF6 protein, which is very similar to TRAF3, indicating the specific binding of iOPN to TRAF3. Similar to our study, Inoue *et al*. recently reported iOPN interacts with TNF receptor-associated factor 2 (TRAF2) in CD40 and TLR4 signaling pathways to regulate TNF production by LPS-stimulated macrophages and to control LPS-induced endotoxemia^38^.

We further demonstrated that iOPN inhibits K48-linked TRAF3 polyubiquitination and degradation. Based on these data, we proposed that binding of iOPN to TRAF3 preventing an E3 ligase from binding to and ubiquitnating TRAF3. Traid3A has been reported to be an E3 ligase involved in TRAF3 ubiquitination and degradation after virus infection^22^. Indeed, we found Triad3A-mediated K48-linked ubiquitination and degradation of TRAF3 was greatly attenuated by iOPN expression. Recently, ubiquitin specific protease 25 (USP25) has been reported to cleave K48-linked ubiquitination to stabilize TRAF3^30^,^31^. We found overexpression of USP25 indeed decreased TRAF3 K48-linked ubiquitination. But, iOPN could not further decrease K48-linked ubiquitination of TRAF3 (Fig. 6A). Thus, iOPN may mainly prevent Triad3A from binding to and ubiquinating TRAF3, leading to its stabilization.

Cantor and colleagues showed that intracellular osteopontin (iOPN) plays essential roles in the differentiation of Follicular helper T cells (T_FH_ cells) and follicular regulatory T cells (T_FR_ cells)^39^. Mechanistically, they demonstrated that iOPN translocates into nucleus and interacts with transcription factor Bcl-6 after activation of the signaling via the receptor ICOS. Binding of iOPN prevented Bcl-6 from ubiquitination-mediated degradation. Similarly, we found iOPN bound to TRAF3 and prevented its degradation mediated through K48-linked ubiqutiation. Therefore, iOPN may bind to and stabilize key molecules in various signaling pathways to regulate the immune responses.

In conclusion, we identified a novel function for iOPN to positively regulate the production of IFN-β and the antiviral response. Our study also delineated a new regulatory mechanism in innate antiviral signaling through iOPN-mediated stabilization of TRAF3. Therefore, iOPN is a very important regulatory component in the antiviral response and may represent a new target for drug design against virus infection.

## Methods

### Cells and reagents

HEK293 and A549 cells were obtained from American Type Culture Collection (Manassas, VA). Mouse primary peritoneal macrophages were prepared from Female C57BL/6J mice (5–6 weeks old) through intraperitoneal injection with thioglycolate. The cells were cultured at 37 °C under 5% CO_2_ in DMEM supplemented with 10% FBS (Invitrogen-Gibco), 100 U/ml penicillin, and 100 μg/ml streptomycin. MG132 and LPS (Escherichia coli, 055:B5) were purchased from Sigma (St. Louis, MO). poly(I:C), cGAMP and ISD were purchased from Invivogen (San Diego, CA, USA). LPS, poly(I:C) and ISD were used at a final concentration of 100 ng/ml, 10 μg/ml and 10 μg/ml, respectively. The antibodies specific to Myc (9B11, #2276), IRF-3 (D83B9, #4302), phospho-IRF3 at Ser^396^ (4D4G, #4947), PCNA (PC10, #2586) and TBK1 (D1B4, #3504) were from Cell Signaling Technology (Beverly, MA). The antibody for Flag (F3165) and VSV G protein (V4888) were from Sigma-Aldrich (St. Louis, MO). The antibody for OPN (AKm2A1, sc-21742) for immunoprecipitation (IP) and western blot (WB) was from Santa Cruz Biotechnology (Santa Cruz, CA). The neutralizing OPN antibody (AF808) was from R&D Systems (Minneapolis, MN). The antibody for HA was from Beijing MDL Biotechnology (Beijing, China). The antibody for TRAF3 (EP1730Y, ab76147) and antibody for ubiquitin-k48 (linkage-specific K48) (EP8589, ab140601) were purchased from Abcam (Cambridge, MA). Their respective horseradish peroxidase-conjugated secondary antibodies were from Santa Cruz Biotechnology (Santa Cruz, CA). Sendai virus was purchased from China Center for Type Culture Collection (Wuhan University, China). Vesicular stomatitis virus (VSV) and HSV-1 were provided by Professor Lizeng Qin (Institute of Basic Medicine, Shandong Academy of Medical Sciences, China).

### Mice

WT C57BL/6J mice were obtained from Joint Ventures Sipper BK Experimental Animal (Shanghai, China). *Spp1*^−/−^ mice (B6.Cg-Spp1tm1blh/J; cat. no. 004936) in the C57BL/6J background are provided by Prof. Zhinan Yin (Jinan University, Guangzhou, China), who originally obtained the mice from The Jackson Laboratory. OPN-deficient mice were backcrossed with WT C57BL/6J mice for 7 generations. *Spp1*^+/−^ heterozygous mice were bred to generate age-matched *Spp1*^+/+^ and *Spp1*^−/−^ mice for experiments in Fig. 1. For other experiments, *Spp1*^−/−^ homozygous mice were mated to generate OPN-deficient mice. Age- and sex-matched WT C57BL/6J littermates were used as controls. Mice were hosted in a pathogen-free facility under standard 12-hour light-dark cycle, fed standard rodent chow, and water ad libitum. All animal experiments were undertaken in accordance with the National Institute of Health Guide for the Care and Use of Laboratory Animals, with the approval of the Scientific Investigation Board of Medical School of Shandong University, Jinan, Shandong Province, China.

### Sequences, plasmid constructs, and transfection

Murine full-length OPN cDNA was cloned by standard RT-PCR and inserted into the pFLAG-CMV2 vector (Sigma-Aldrich) with the following primers: forward, 5′-CCGGAATTCAATGAGATTGGCAGTGATTTG-3′; reverse, 5′-CGCGGATCCTTAGTTGACCTCAGAAGATGAACTC-3′. The expression plasmid for intracellular form OPN (iOPN) was constructed by deleting the amino acids from 1 to 16 of OPN and inserted into the pFLAG-CMV2 vector with the following primers: forward, 5′-CCGGAATTCACTCCCGGTGAAAGTG-3′; reverse, 5′-CGCGGATCCTTAGTTGACCTCAGAAGATGAACTC-3′. The amino acid sequences of full length OPN and iOPN were shown in Supplementary Table S1. IFN-β promoter reporter and IFN-β PRD I/III reporter were provided by Dr. Katherine A. Fitzgerald (University of Massachusetts Medical School, MA). TRAF3 and TRAF6 expression plasmids were provided by Dr. Michael Karin (University of California at San Diego, CA). Expression plasmid for USP25 was provided by Dr. Bo Zhong (Wuhan University, China). Expression plasmids for RIG-I, MDA5, MAVS, TBK1, IRF3 and HA-Ub were used as described^40^. Triad3A expression plasmid was purchased from Vigene Biosciences (Jinan, China). The IRF3 5D was constructed by replacing the five residues at position 396, 398, 402, 404, and 405 of IRF3 with phosphomimetic aspartate amino acid. iOPN-WT, iOPN-N, iOPN-C sequence were obtained from iOPN expression plasmid by RT-PCR, TRAF3 or Triad3A sequence were obtained from TRAF3 expression plasmid or Triad3A expression plasmid by RT-PCR, these sequences were cloned into pcDNA3.1-HisC vector (invitrogen) for expression of indicated proteins by using *in vitro* Transcription/Translation System. For transient transfection of plasmids into HEK293 and A549 cells, X-treme Gene 9 reagents were used (Roche). For transient silencing, duplexes of siRNA were transfected into peritoneal macrophages with the GenePORTER 2 Transfection Reagent (Genlantis) according to the standard protocol. Target sequences for transient silencing were 5′-GGACUGAGGUCAAAGUCUATT-3′ (siRNA) for OPN, and scrambled control sequences were 5′-UUCUCCGAACGUGUCACGU-3′.

### Immunoprecipitation and western blot analysis

For immunoprecipitation (IP), whole-cell extracts were prepared by lysing the cells in IP buffer (1.0% (vol/vol) Nonidet P-40, 50 mM Tris-HCl pH7.4, 50 mM EDTA, 150 mM NaCl) adding protease inhibitor cocktail (Merck). After centrifugation for 10 min at 14,000 × *g*, supernatants were collected and protein concentrations in the extracts were measured with a bicinchoninic acid assay (Pierce, Rockford, IL) and were made equal with extraction reagent. For IP, the supernatants were incubated with protein G plus-agarose immunoprecipitation reagent (Santa Cruz) together with 1μg monoclonal anti-Flag or 1 μg anti-Myc. After 6 h of incubation, beads were washed five times with IP buffer. Immunoprecipitates were eluted by boiling with 1% (wt/vol) SDS sample buffer. For western blot analysis, immunoprecipitate or whole-cell lysates were loaded, subjected to SDS-PAGE, transferred onto nitrocellulose membranes, and then blotted with specific antibodies. Nuclear proteins and cytoplasmic proteins were extracted by NE-PER Protein Extraction Reagent (Pierce) according to the manufacturer’s instructions.

### ELISA

The concentrations of IFN-β in culture supernatants and sera were measured by ELISA Kits Legend Max (Biolegend, San Diego, CA). The concentrations of sOPN in culture supernatants were measured by a ELISA kits from R&D Systems (Minneapolis, MN).

### RT-PCR and qRT-PCR assays

Total RNA was extracted with TRIzol reagent according to the manufacturer’s instructions (Invitrogen). Specific primers used for RT-PCR and qRT-PCR assays were 5′-GCCGAGGTGATAGCTTGGCTTAT-3′, 5′-ATGGCTGCCCTTTCCGTTGT-3′ for m-OPN, 5′-AGTTACACTGCCTTTGCC-3′, 5′-GTTGAGGACATCTCCCAC-3′ for m-IFN-β, 5′-ATGTGGCAGAGGGAGAATGTC-3′, 5′-CAAACCCTGGCAATTCTCGT-3′ for m-MX1, 5′-CCTCACCATCATCCTCACTG-3′, 5′-AAACACGACTGCAAGATTGG-3′ for m-CCL5, 5′-CCGTCATTTTCTGCCTCATC-3′, 5′-GTGGCAATGATCTCAACACG-3′ for m-CXCL10, 5′-TGTTACCAACTGGGACGACA-3′, 5′-CTGGGTCATCTTTTCACGGT-3′ for m-Actin. Reverse transcription was performed using FastQuant RT Kit (with DNase) (Tiangen Biotech). The qPCR was performed using Roche LightCycler 480 Real-Time PCR system. Actin was used as the internal control, and the 2^−ΔΔCT^ method^41^ was used to evaluate the relative quantities of each amplified product in the samples. The results were normalized to actine. For each qPCR analysis, three technical replicates were performed.

### Ubiquitination assays

For analysis of the ubiquitination of TRAF3 in HEK293 cells, HEK293 cells were transfected with Myc-TRAF3, HA-Ub (WT), or HA-Ub mutants and Flag-iOPN or Triad3A, and then whole-cell extracts were prepared in IP buffer and immunoprecipitated with anti-Flag followed by immunoblot with anti-HA antibody. For analysis of endogenous TRAF3 ubiquitination in macropahges, whole-cell extracts were prepared in IP buffer and immunoprecipitated with anti-TRAF3 antibody followed by immunoblot with anti-ubiquitin antibody. To exclude the detected TRAF3 ubiquitination may be from TRAF3-interacting proteins, IP under stringent conditions was performed. Briefly, cell lysates were first prepared with denaturing buffer (50 mM Tris pH7.4, 140 mM NaCl, 1% SDS), boiled for 5 min and then diluted 10-fold with buffer containing 50 mM Tris pH7.4, 140 mM NaCl, 1% Triton X-100, and protease inhibitor mixture (Merck). Samples were centrifuged at 16,000 × g for 10 mins. Cell lysates were then immunoprecipitated with anti-TRAF3 antibody and analyzed by immunoblot with anti-Ubiquitin antibody.

### Assay of luciferase activity

Luciferase activity was measured with the Dual-Luciferase Reporter Assay system according to the manufacturer’s instructions (Promega) as described^42^. Data were normalized for transfection efficiency by division of firefly luciferase activity with that of renilla luciferase.

### *In vitro* binding and ubiquitination assay

Recombinant TRAF3, Triad3A, iOPN-WT, iOPN-N, iOPN-C proteins were made with a TNT Quick Coupled Transcription/Translation System (Promega) according to the instructions of the manufacturer. Briefly, reaction mixture contained 1ug indicated plasmid DNA and 1mM methionine was incubated at 30 °C for 60–90 minutes, and WB was performed for analyzing the results of translation. Binding assays were performed by mixing TRAF3, Triad3A and iOPN-WT protein together, followed by IP with TRAF3 antibody and WB with OPN antibody. Ubiquitination was analyzed with an ubiquitination kit (Boston Biochem) following protocols recommended by the manufacturer. Recombinant proteins were mixing with 100 nM E1, 2 mM E2 and 200 mM Ub-K48 in a final volume of 20 ml reaction buffer (50 mM Hepes pH 8.0, 100 mM NaCl, 10 mM Mg^2+^ −ATP, 0.5 mM DTT). The reaction was carried out at 37 °C for 1 h and products were analyzed by immunoblotting with anti-TRAF3 antibody.

### Native PAGE

The IRF3 dimerization assay was performed as described previously with modifications^43^. In brief, macrophages were harvested with 30 ml of ice-cold lysis buffer (50 mM Tris/HCl, pH 7.5, 150 mM NaCl, and 0.5% NP-40) containing protease inhibitor cocktail. After centrifugation at 13,000 g for 10 min, supernatants were quantified using a BCA assay (Thermo Fisher Scientific) and diluted with 2× native PAGE sample buffer (125 mM Tris/HCl, pH 6.8, 30% glycerol, and 0.1% Bromophenol blue), then 20 μg of total protein was applied to a pre-ran 7.5% native gel for separation. After electrophoresis, the proteins were transferred onto a nitrocellulose membrane for immunoblotting.

### VSV infection of mice

For *in vivo* cytokine production studies, *Spp1*^−/−^ and WT mice (female, 6–8 weeks old) were intraperitoneally infected with VSV (1 × 10^8^ pfu per mouse). The virus RNA in lung, spleen and liver were determined by qRT-PCR and by measurement of VSV V protein with VSV-G antibody. For the survival experiments, mice were intravenously infected with VSV (5 × 10^8^ pfu per mouse) and then monitored for survival after VSV infection.

### VSV plaque assay and detection of virus replication

VSV plaque assay was performed as previously described^40^. The HEK293 cells (2 × 10^5^) were transfected with the indicated plasmids for 36 h before VSV infection (MOI of 0.1). At 1 h after infection, cells were washed with PBS three times and then medium was added. The supernatants were harvested 24 h after washing. The supernatants were diluted 1:10^6^ and then used to infect confluent HEK293 cells cultured on 24-well plate. At 1 h after infection, the supernatant was removed, and 3% methylcellulose was overlaid. At 3 d after infection, overlay was removed, and cells were fixed with 4% formaldehyde for 20 min and stained with 0.2% crystal violet. Plaques were counted, averaged, and multiplied by the dilution factor to determine viral titer as LOG10 (pfu/ml). Total HEK293 cellular RNA was extracted and VSV RNA replicates were examined by qRT-PCR. Primers for VSV were as follows: 5′-ACGGCGTACTTCCAGATGG-3′ (sense) and 5′-CTCGGTTCAAGATCCAGGT-3′ (antisense).

### Lentivirus preparation and infection

Lentiviral expression plasmids for iOPN-WT, iOPN-N, iOPN-C were constructed by inserting the corresponding coding sequence into pWPXL vector (addgene). Lentivirus particles were produced through transfection of pWPXL-OPN, psPAX2 and pMD2.G plasmids (addgene) with a proportion of 20:15:7 into HEK293T cells, 3 days later the culture was harvested and enriched by PEG8000. The enriched lentivirus particle (MOI, 50) was used to infect WT and OPN-deficient macrophages for 4 days.

### Statistical analysis

All experiments were performed for three or more times with similar results. The data were presented as mean ± SD of one representative experiment. Statistical significance was determined with the two-tailed Student’s *t* test, with a P value of <0.05 considered statistically significant.

## Additional Information

**How to cite this article**: Zhao, K. *et al*. Intracellular osteopontin stabilizes TRAF3 to positively regulate innate antiviral response. *Sci. Rep*. **6**, 23771; doi: 10.1038/srep23771 (2016).

## Supplementary Material

Supplementary Information
