# Supplementary material for: Intracellular osteopontin stabilizes TRAF3 to positively regulate innate antiviral response
Source: Sci Rep. 2016 Mar 30;6:23771. doi: 10.1038/srep23771 (PMC4824456; doi:10.1038/srep23771)
Supplement: Supplementary Information [file srep23771-s1.pdf]

Supplementary Information

Title of manuscript:
Intracellular osteopontin stabilizes TRAF3 to positively regulate innate antiviral response

Authors:
Kai Zhao, Meng Zhang, Lei Zhang, Peng Wang, Guanhua Song, Bingyu Liu, Haifeng Wu, Zhinan Yin, Chengjiang Gao*.

Supplementary information includes:
Supplementary Figures S1-S4 and Table S1

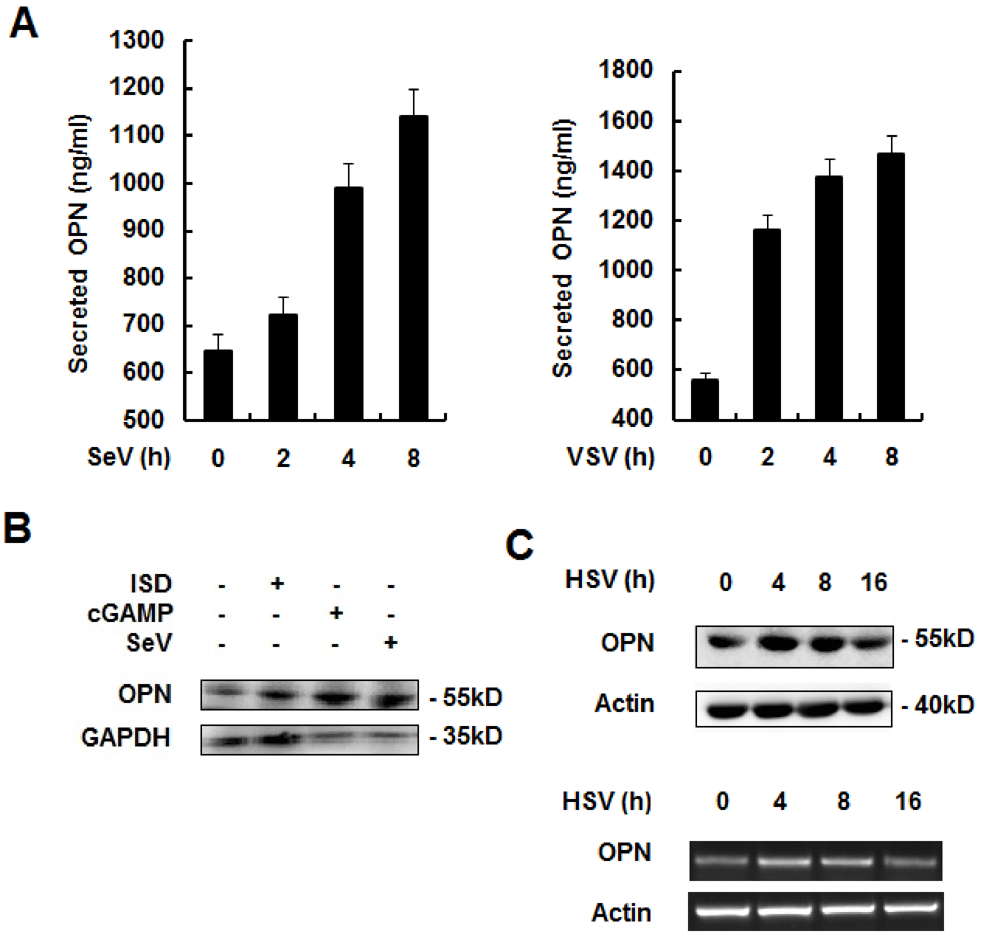

Figure S1. OPN expression is induced upon virus infection. (**A**) ELISA analysis of secreted OPN in mouse peritoneal macrophages infected with SeV and VSV for indicated times. (**B**) Western blot of OPN protein in mouse peritoneal macrophages transfected with ISD and cGAMP for 8 h. (**C**) Western blot and RT-PCR analysis of OPN expression in mouse peritoneal macrophages infected with HSV-1 for indicated times. Data are representative of three independent experiments (mean ±S.D. in A).

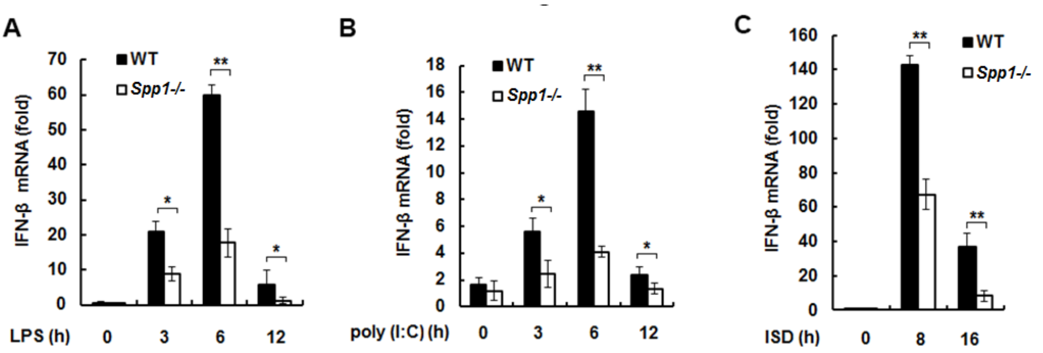

Figure S2. OPN positively regulates LPS-, poly(I:C)- and intracellular DNA-induced IFN-β production. (A-C) Quantitative RT-PCR analysis of IFN-β mRNA in peritoneal macrophages from WT mice or *Spp1*$^{-/-}$ mice stimulated with LPS (A), poly(I:C) (B) or ISD (C) for indicated times. Data are representative of three independent experiments (mean ±S.D. in A-C).

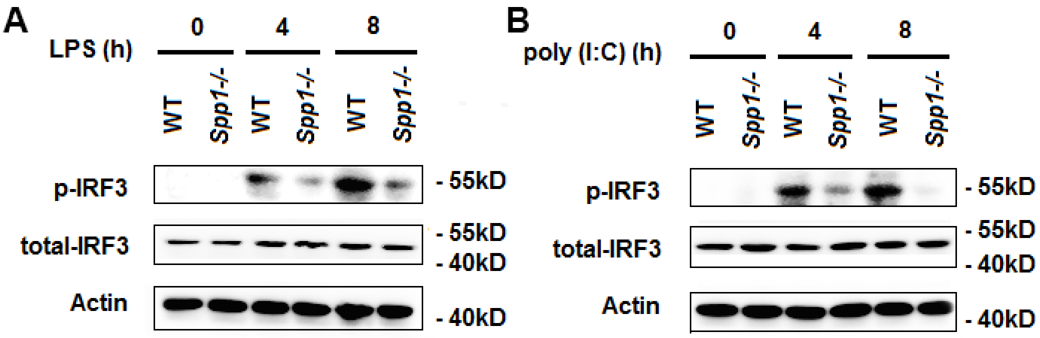

Figure S3. OPN positively regulates TLR3/4-induced IRF3 activation. (A-B) Western blot of phosphorylated IRF3 and total IRF3 in WT and OPN-deficient (*Spp1*$^{-/-}$) peritoneal macrophages stimulated with LPS (A) or poly(I:C) (B) for indicated times. Data are representative of three independent experiments.

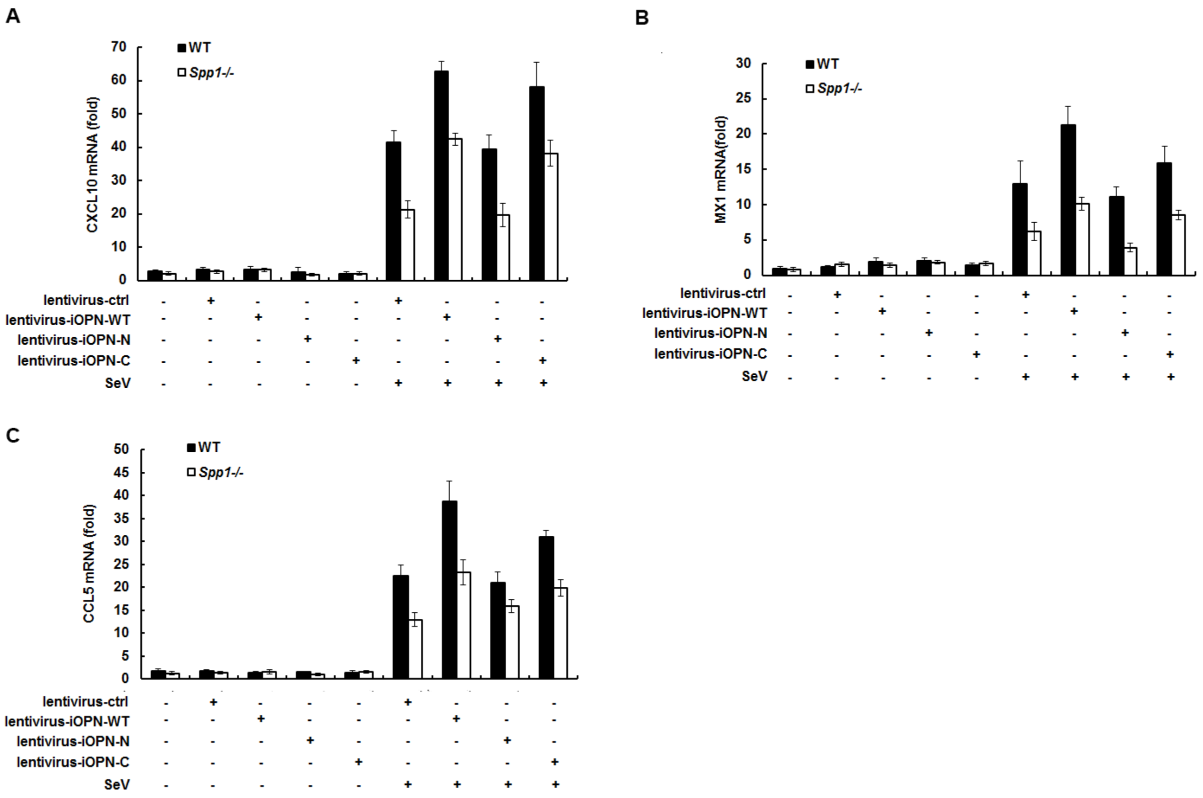

Figure S4. Transfection of OPN expression plasmids restored ISGs expression in OPN-deficient macrophages. (A-C) Peritoneal macrophages from WT and OPN-deficient (*Spp1*$^{-/-}$) mice were infected with lentivirus (MOI, 50) containing control vector, iOPN-WT, iOPN-N or iOPN-C for 4 days, then infected with SeV for 8 h or left uninfected, quantitative RT-PCR analysis of CXCL10 (A), Mx1 (B) and CCL5 (C) were performed. Data are representative of three independent experiments (mean ± S.D. in A-C).

Table S1. Amino acid sequence information of iOPN and full length OPN expression plasmid.

iOPN expression plasmid (amino acid sequence information)				
----------	------LPVK	VTDSGSSEEK	LYSLHPDPIA	TWLVPDPSQK
QNLLAPQNAV	SSEEKDDFKQ	ETLPSNSNES	HDHMDDDDDD	DDDDGDHAES
EDSVDSDESD	ESHHSDESDE	TVTASTQADT	FTPIVPTVDV	PNGRGDSLAY
GLRSKSRSFQ	VSDEQYPDAT	DEDLTSHMKS	GESKESLDVI	PVAQLLSMPS
DQDNNGKGSH	ESSQLDEPSL	ETHRLEHSKE	SQESADQSDV	IDSQASSKAS
LEHQSHKFHS	HKDKLVLDPK	SKEDDRYLKF	RISHELESSS	SEVN
Full length OPN expression pladmid (amino acid sequence information)				
MRIAVICFCL	FGIASSLPVK	VTDSGSSEEK	LYSLHPDPIA	TWLVPDPSQK
QNLLAPQNAV	SSEEKDDFKQ	ETLPSNSNES	HDHMDDDDDD	DDDDGDHAES
EDSVDSDESD	ESHHSDESDE	TVTASTQADT	FTPIVPTVDV	PNGRGDSLAY
GLRSKSRSFQ	VSDEQYPDAT	DEDLTSHMKS	GESKESLDVI	PVAQLLSMPS
DQDNNGKGSH	ESSQLDEPSL	ETHRLEHSKE	SQESADQSDV	IDSQASSKAS
LEHQSHKFHS	HKDKLVLDPK	SKEDDRYLKF	RISHELESSS	SEVN
